# Radiopaque orbital foreign bodies from retained infant brachytherapy seeds

**DOI:** 10.1016/j.ajoc.2026.102693

**Published:** 2026-10-23

**Authors:** Megan Jung, Kapil Mishra, Lilangi S. Ediriwickrema

**Affiliations:** a University of California, Irvine, School of Medicine, Irvine, CA, 92697, USA; b Gavin Herbert Eye Institute, Department of Ophthalmology, University of California, Irvine, 92697, USA

**Keywords:** Brachytherapy, Foreign bodies, Multidisciplinary care, Ocular oncology, Ophthalmology, Orbit, Radon

## Abstract

**Purpose:**

The purpose of this case report is to describe a rare case of retained orbital brachytherapy seeds implanted during infancy and identified in an older adult undergoing neurocognitive evaluation. It highlights a multidisciplinary approach to diagnosis and management.

**Observations:**

An 85-year-old man presented for removal of radiopaque periocular foreign bodies prior to magnetic resonance imaging (MRI) for a neurocognitive workup. The patient reported undergoing “seed placement” radiation therapy to the right orbital region at 18 months of age in Scotland for a superior right orbital mass, suspected to be a capillary hemangioma. Computed tomography revealed two discrete radiopaque foreign bodies in the superior right orbit and right medial nasal soft tissue. Multidisciplinary evaluation with ocular oncology and radiation safety suggested that the foreign bodies were retained casings from historical radon-222 brachytherapy seeds. Preoperative and intraoperative assessments demonstrated no detectable residual radioactivity, and the foreign bodies were removed successfully, allowing the patient to proceed with MRI.

**Conclusions and importance:**

This case highlights the importance of recognizing historical radiation treatments as potential causes of orbital foreign bodies and imaging artifacts in older adult patients. It also underscores the value of multidisciplinary management for the safety of patients.

## 1 Case presentation

An 85-year-old man with a prior history of resected cutaneous melanoma, proctectomy due to prostate cancer, primary open angle glaucoma, and recently diagnosed mild cognitive impairment was referred to the oculoplastics service for evaluation of life-long radiopaque foreign bodies along the right periocular region. The foreign bodies were requested to be removed prior to proceeding with magnetic resonance imaging (MRI) for neurocognitive workup and evaluation by his neurologist. On further questioning, the patient reports a history of radiation therapy via “seed placement” to the right orbital region at 18 months of age in 1940 for a right superior orbital mass, while residing in Scotland. Childhood photographs reveal a right superior orbital mass (Fig. 1A), raising suspicion for a capillary hemangioma; however, the original medical records have become inaccessible.

**Fig. 1:**
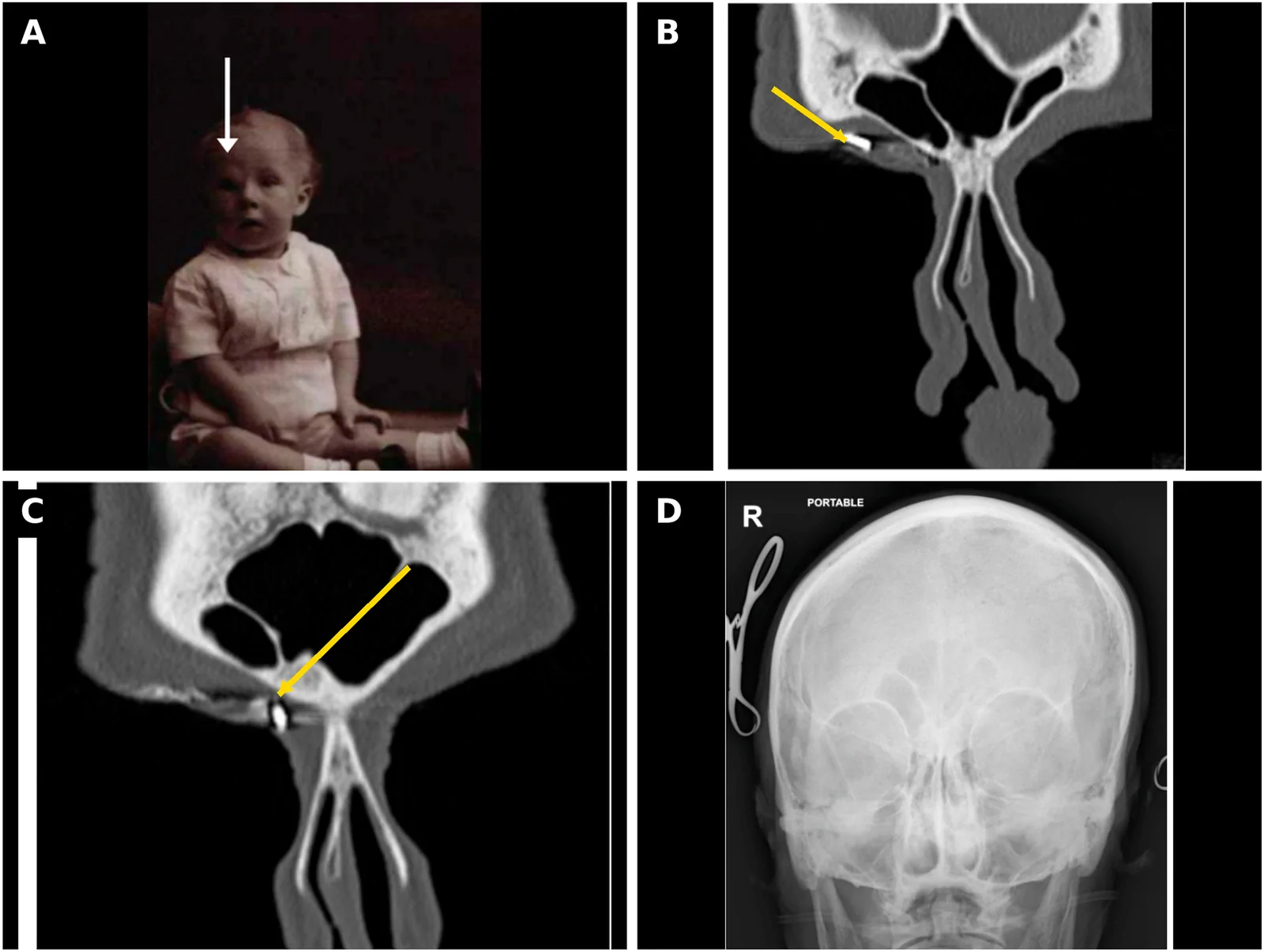
An 85-year-old man with retained radiopaque foreign bodies and a history of childhood orbital radiation using “seed placement.” (A) Childhood photograph demonstrating a right superior orbital mass before radiation treatment. Preoperative computed tomography demonstrating radiopaque foreign bodies (arrows) in the (B) superior right orbit and (C) right medial nasal region. (D) Postoperative radiography confirming complete removal of the foreign bodies.

The patient denied vision loss or ocular discomfort. Examination revealed stable best corrected visual acuity of 20/30 in both eyes with intraocular pressures of 14 mmHg in the right eye (OD) and 12 mmHg in the left eye (OS). Glaucoma medications administered in both eyes included travoprost 0.004% nightly and dorzolamide-timolol preservative-free 2%-0.5% twice daily. Dilated fundus examination was unremarkable bilaterally. Pre-operative computed tomography scan of the face revealed two discrete radiopaque foreign bodies. One, approximately 0.7 cm, was found in the superior right orbit below the orbital rim at the 11:00 position (Fig. 1B). A second, approximately 0.6 cm, was found within the right medial nasal soft tissue (Fig. 1C).

Ocular oncology and radiation safety specialists were consulted before surgical intervention. Preoperative assessment demonstrated no detectable radioactivity, and radiation safety personnel determined that no additional shielding precautions were required. The patient subsequently underwent right orbitotomy with exploration and removal of the foreign bodies by the oculoplastics team (Fig. 2). Intraoperative radiation monitoring demonstrated no detectable residual radioactivity. Both foreign bodies were removed intact, and postoperative radiography confirmed complete removal (Fig. 1D). Definitive compositional analysis was not available; however, based on the patient's history and historical treatment practices, the foreign bodies were presumed to represent radon-based brachytherapy seeds. The patient had an uncomplicated postoperative course and was able to proceed with MRI for neurologic evaluation.

**Fig. 2:**
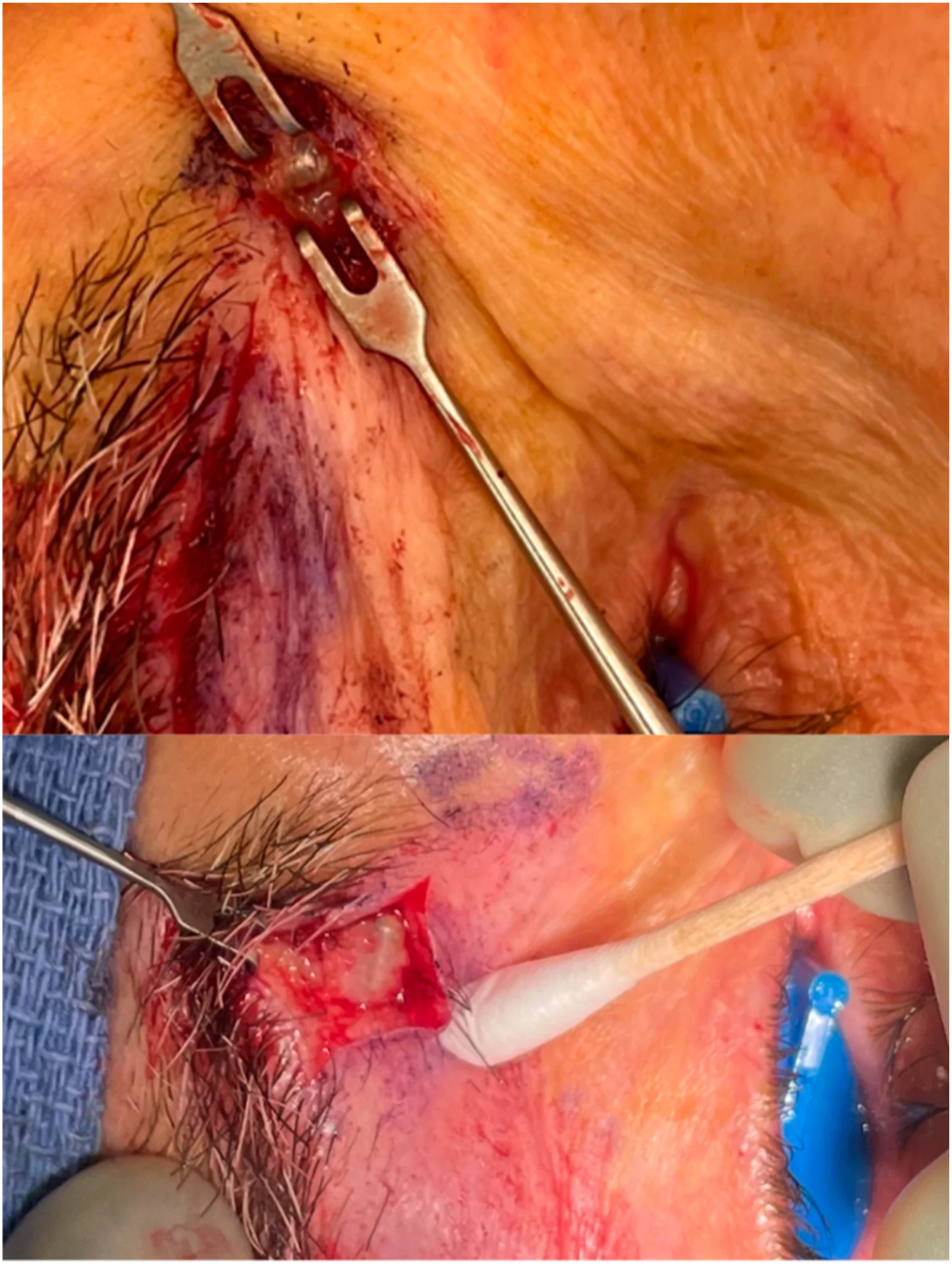
Intraoperative removal of the presumed radon brachytherapy seeds from the right periocular (top) and nasal (bottom) region in an 85-year-old man with a history of childhood “seed placement” orbital radiation for presumed capillary hemangioma.

## 2 Discussion

Given the patient's clinical history and imaging findings, the diagnosis of this patient is concerning for retained brachytherapy seeds, possibly containing radon-222. Historically, these seeds were used for ocular/orbital lesions, including uveal melanomas or capillary hemangiomas.^1^, ^2^ Radon seeds were generally intended to remain in situ after implantation while their radioactive contents decayed rather than being routinely removed after treatment. Therefore, retention of the presumed seeds in this patient was consistent with historical treatment practices.^1^ However, removal may be indicated in modern practice for diagnostic clarification or to permit MRI. Although the radioactive half-life of radon-222 is 3.8 days, the metal casings of radon seeds were typically made of gold, which can remain detectable on imaging decades later and appear radiopaque.^3^, ^4^

Management of retained orbital brachytherapy seeds should be individualized according to the patient's symptoms, need for subsequent imaging or treatment, anatomic location of the foreign bodies, and uncertainty regarding residual radioactivity. Although retained seeds may pose minimal radiation risk decades after implantation, they may produce imaging artifacts, mimic pathologic findings, or complicate clearance for MRI when their composition and magnetic properties are uncertain.^5^ Consultation with oculoplastics, ocular oncology, and radiation safety can help determine whether removal is necessary and whether radiation monitoring or additional operative precautions are required. When removal is pursued, preoperative imaging can help localize the foreign bodies and guide the surgical approach.

In this case, removal was indicated to permit MRI for the patient's neurocognitive evaluation. Perioperative testing demonstrated radioactivity below the minimum detectable activity, and no additional shielding precautions were required. Although definitive compositional analysis was unavailable, the patient's history and historical treatment practices supported the presumed diagnosis of retained radon-based brachytherapy seeds.^6^ This case illustrates the importance of recognizing historical radiation treatments in the differential diagnosis of unexplained orbital foreign bodies and of using a multidisciplinary approach when evaluating whether retained brachytherapy implants can be safely observed or should be removed.

## CRediT authorship contribution statement

**Megan Jung:** Writing – original draft. **Kapil Mishra:** Writing – review & editing. **Lilangi S. Ediriwickrema:** Writing – review & editing.

## Patient consent

Written consent to publish this case has been obtained.

## Claim of priority

After conducting a literature review on September 16, 2026, utilizing PubMed and Google Scholar with the keywords “orbital brachytherapy, radon seeds, radon-222, retained orbital foreign body, eyelid radon seed, orbit radon seed extrusion”, we did not identify prior reports describing retained orbital radon-222 brachytherapy seeds implanted in infancy and surgically removed more than eight decades later to facilitate magnetic resonance imaging.

## Authorship

All authors attest that they meet the current ICMJE criteria for Authorship.

## Artificial intelligence statement

No artificial intelligence (AI) tools were used in the preparation of this manuscript.

## Declaration of competing interest

The authors declare the following financial interests/personal relationships which may be considered as potential competing interests: Research to Prevent Blindness unrestricted grant to UCI Ophthalmology.Megan Jung reports was provided by University of California Irvine School of Medicine. If there are other authors, they declare that they have no known competing financial interests or personal relationships that could have appeared to influence the work reported in this paper.
